# An Effective Ostrich Oil Bleaching Technique Using Peroxide Value as an Indicator

**DOI:** 10.3390/molecules16075709

**Published:** 2011-07-05

**Authors:** Uma Devi Palanisamy, Muniswaran Sivanathan, Ammu Kutty Radhakrishnan, Nagaraja Haleagrahara, Thavamanithevi Subramaniam, Gan Seng Chiew

**Affiliations:** 1 School of Medicine and Health Sciences, Monash University, Sunway Campus, 46150, Malaysia; 2 Faculty of Medicine and Health, International Medical University, 57000, Kuala Lumpur, Malaysia; E-Mails: Padmasriiswar@yahoo.com (M.S.); ammu_Radhakrishnan@imu.edu.my (A.K.R.); hsnagaraja@gmail.com (N.H.); 3 SIRIM Bhd, 1, Persiaran Dato Menteri, 40911, Shah Alam, Selangor Darul Ehsan, Malaysia; E-Mail: thava@sirim.my (T.S.); 4 Allergy and Immunology Research Centre, Institute for Medical Research, 50586, Kuala Lumpur, Malaysia; E-Mail: gansc@imr.gov.my (G.S.C.)

**Keywords:** crude ostrich oil, temperature, time, peroxide value, clay

## Abstract

Ostrich oil has been used extensively in the cosmetic and pharmaceutical industries. However, rancidity causes undesirable chemical changes in flavour, colour, odour and nutritional value. Bleaching is an important process in refining ostrich oil. Bleaching refers to the removal of certain minor constituents (colour pigments, free fatty acid, peroxides, odour and non-fatty materials) from crude fats and oils to yield purified glycerides. There is a need to optimize the bleaching process of crude ostrich oil prior to its use for therapeutic purposes. The objective of our study was to establish an effective method to bleach ostrich oil using peroxide value as an indicator of refinement. In our study, we showed that natural earth clay was better than bentonite and acid-activated clay to bleach ostrich oil. It was also found that 1 hour incubation at a 150 °C was suitable to lower peroxide value by 90%. In addition, the nitrogen trap technique in the bleaching process was as effective as the continuous nitrogen flow technique and as such would be the recommended technique due to its cost effectiveness.

## 1. Introduction

Ostrich oil has been used for centuries by the Egyptian, Roman and African cultures for topical relief of dry skin, burns, lesions, contact dermatitis, eczema, psoriasis, sunburn, chapped lips, muscular pain, hair growth, dry hair, bed sores, fine lines and wrinkles, to soften cracked heels and for minor cuts and scratches. Ratite oils are used extensively in the cosmetics and pharmaceutical industry. They are reputed to have exceptional moisturizing, penetrating and therapeutic qualities for humans and animals. Emu oil, for instance, has been shown to have anti-inflammatory and possibly skin de-sensitizing properties [1].

Ostrich oil is rich in polyunsaturated fatty acids (PUFA). There are many reports on the effects of PUFAs in terms of their ability to modify cell membrane phospholipids, modify cellular functions, exert a protective role towards normal tissues and low cytotoxicity to normal cells [2]. Ostrich oil contains omega 9 (oleic), omega 6 (linoleic) and omega 3 (linolenic), essential fatty acids (EFAs) and certain vitamins and amino acids that help maintain the health of skin membranes [3]. Ostrich oil has the ability to penetrate deeply into the skin, unlike petroleum based products and its non-comodegenic property provides moisture for hours without clogging pores. This is due to its high levels of oleic acid [4] and as such can be used as a carrier agent in combination with various medicinal or cosmetic ingredients and to deliver them beneath the skin barrier [5].

EFAs have been known to ensure the proper functioning of the cardiovascular, reproductive, immune and nervous systems. These EFAs are used in the production of phospholipids that are necessary for the formation and maintaining the integrity of healthy cell membranes, neuronal development and functioning of the brain and nervous system [6,7]. EFAs are nutritionally important because they act as precursors to a group of hormone-like substances called eicosanoids which comprise of prostaglandins, thromboxanes and prostacyclins that help in regulating the central nervous system, blood pressure, heart rate and play a role in the immune system by regulating inflammation and encouraging our body to fight infections [8].

Omega-6 fatty acids are generally necessary for skin and growth development, regulating metabolism, promoting transport of fatty acids from liver to the tissues and maintaining reproductive performance. These fatty acids have become increasingly popular in the cosmetic industry due to its beneficial properties on the skin [9]. Research has shown that linoleic acid, when applied topically on the skin, has anti-inflammatory, acne reduction and moisture retention properties [10]. It has been shown that EFA deficiency which induces inflammatory processes in rats and humans can be reversed by the cutaneous application of linoleic acid [11]. Meanwhile, omega-3 fatty acids are beneficial for reducing hypertension [12,13] and stroke risk, [14] decreasing effect of arthritis [15,16], increasing autoimmune disease survival rates, aiding in prevention of cancer [17] and many more. Omega 9, on the hand plays a role in inhibiting breast cancer and promotes healthy inflammation responses. Omega 9 may also aid in the production of prostaglandins, which has many health benefits [18]. 

However, rancidity causes undesirable chemical change in these PUFA’s. Lipid peroxidation is considered the main cause of oil rancidity. Peroxidation is more common in oils which are rich in PUFA such as ostrich, emu and rhea oil. In addition, rancidity also promotes the formation of free radicals such as hydroxyl and peroxyl radicals which are reported to be associated with mutagenesis, carcinogenesis and aging [11]. Free fatty acids formed further deteriorate into peroxides which then decompose into odorous material which turns rancid. Rancidity in oils causes a change in flavour, colour, odour and its nutritional value [19]. Oxidative rancidity is the main reason oils are rejected by consumers [20]. The purpose of bleaching is to remove all the impurities from the ostrich oil without removing or damaging any of the beneficial properties [21].

A known method used to refine rhea oil [22], where the rendered oil is heated to 160 degrees F, after which, 1-2% diatomaceous earth is added by weight to the preheated oil and oil is held at 200 degrees F, under vacuum and with agitation, for 5 minutes. The oil is vacuum filtered twice to remove all the bleaching earth and to separate the solid fat from the pure oil. The refined oil is then cooled in stages to 98 degrees F for 24 hours, 74 degrees F for 24 hours, and 60 degrees F for 24 hours 

Another bleaching process for ostrich oil [23] is by adding the minced fat mass into a steaming cooker and oil is separated with 60-90 °C hot water. Activated clay is added to the crude ostrich oil, which is agitated at 70-80 °C for about 10 minutes and filtered by paper filter. Filtered oil is put into a vessel and kept at a state of reduced pressure for more or 1 day to remove odors. Tocopherol (vitamin E) is added to the oil, bamboo charcoal is dipped in the oil in 1/10-1/100 weight of the oil, which is then left standing for 10-20 days to enhance transparency and remove the odors 

The purpose of refining is to remove all the impurities from the ostrich oil without removing or damaging any of the beneficial properties [24]. The objective of this study was to investigate the effects of different types of clays, determine the effective duration and temperature of treatment required to bleach crude ostrich oil. We also compared two different vacuum techniques in their efficiency to bleach the crude ostrich oil.

## 2. Results and Discussion

### 2.1. Ability of Different Types of Clay in Bleaching Crude Ostrich Oil

Natural earth clay, bentonite clay and acid activated clay were used to measure their ability to remove peroxides from crude ostrich oil. Previous studies have shown that acid activated clay is efficient in bleaching crude oil [8,21]. One study using palm oil showed a better removal of the pro-oxidants using acid-activated clay by 90% over neutral clay, where only 80% reduction was observed. [21]. Kheosk, and Lim suggested that the mechanism of effective impurity removal of ostrich oil by acid-activated clay is adsorption of the phosphorus ions on to the lattice structure of the clay [25] while Sarier and Guler stated that the acid activated clay absorbs impurities on its active sites by formation of hydrogen bonds with the coordinated bonds with the Lewis sites [26]. In this study, three different types of clay, natural earth clay, bentonite clay and acid-activated clay were assessed for their ability to bleach crude ostrich oil by measuring the reduction in Peroxide Value. A control experiment without clay was also carried out using the crude ostrich oil. 

**Figure 1:**
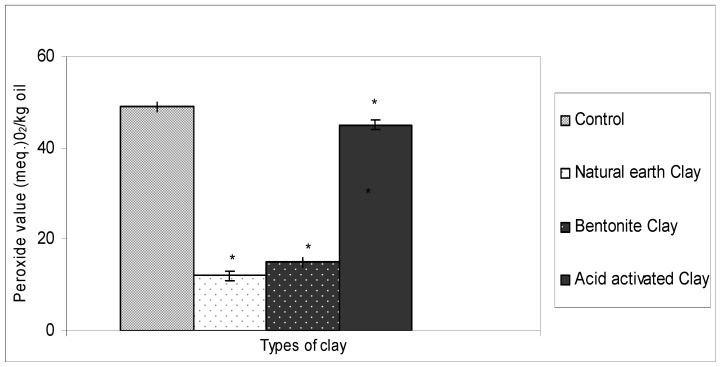
Ability of different types of clay in bleaching crude ostrich oil in removing peroxides.

The results (Figure 1) show that the bleaching ability of the clays varied. It was observed that the natural earth clay and bentonite clay showed a significant reduction in peroxide value when compared to the control. However, acid-activated clay did not show a similar reduction in peroxide value as demonstrated by other workers [25,26]. It has been reported that bentonite and natural earth clay have multiple properties such as swelling, hydration and water adsorption [26].

### 2.2. An Effective Bleaching Time with Bentonite and Natural Earth Clay

The effective time required to bleach crude ostrich oil using bentonite and natural earth clay was investigated. After treating the oil with the respective clays, aliquots of the oils were removed at intervals of 1, 4, 8, 16 and 24 hours and their peroxide values determined.

**Figure 2:**
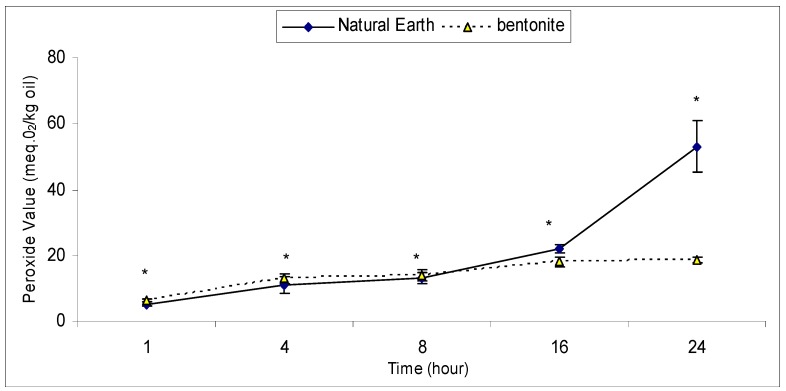
Effective bleaching time of crude ostrich oil using bentonite and natural earth clay in removing peroxides.

The results show that one hour was sufficient to reduce the peroxide value to a desirable level in both clays. As bleaching time increased, peroxide value was observed to have increased correspondingly for the period of this study. It was important to note that only with natural earth clay, we observed a 60% increase in peroxide value from the 16 to the 24^th^ hour (Figure 2). An increase in peroxide formation with time has been reported by Ying and Chun [27]. They explained that the oxidation of oil proceeds with time while adsorption of peroxides ceases due to the capacity of the absorbent. In a similar study by Wang and Lin using soy oil and acid-activated clay, it was observed that peroxide removal efficiency increased steadily from 10 min until its maximum efficiency of 93.9% at 70 min [28]. Our results support these studies indicating that the effective bleaching time to remove peroxides is 1 hour.

### 2.3. Effect of Bleaching Temperature with Bentonite and Natural Earth Clay

**Figure 3:**
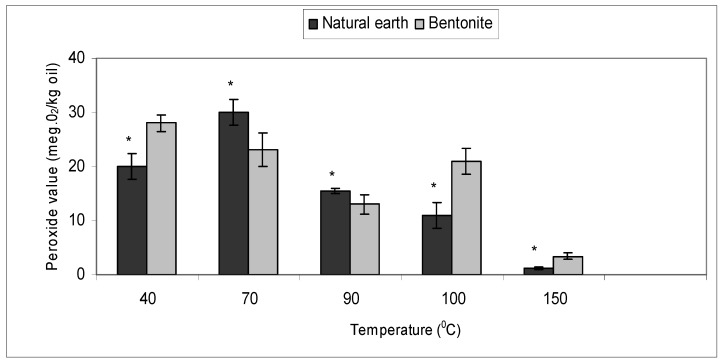
Effect of bleaching temperatures on crude ostrich oil using bentonite and natural earth clay in removing peroxides.

The effective temperature required to bleach crude ostrich oil for 1 hour using bentonite and natural earth clay was studied at 40 °C, 70 °C, 90 °C, 100 °C and 150 °C. The peroxide value at the various temperatures were determined as per described in the experiment (Figure 3).

The results indicated that both bentonite and natural earth clay show a significant difference (*p <* 0.01) in peroxide value at different temperatures. At temperatures above 70 °C, the peroxide value fell below 20, which is the desirable peroxide value of oils intended for therapeutic and cosmetic purposes [29,30]. Studies on various oils have shown that effective bleaching temperature was 110 °C [27] and 120 °C [20] for soy oil and palm oil respectively. In our study, it was observed that bleaching at 150 °C corresponded to a peroxide value of 3 and 1 with bentonite and natural earth clay, respectively. It has been reported that as temperature increases, effectiveness of the clay increases, with the higher temperature even accelerating the rate of decomposition and adsorption of peroxides [28].

To ensure that an increase in temperature does indeed reduce the peroxide value with the addition of natural earth clay indicating that the rate of adsorption is higher than its decomposition the following experiment was carried out. The study was carried out to determine the effect of natural earth clay on bleaching crude ostrich oil as temperature was increased. Controls without the natural earth clay were also carried out at the various temperatures.

**Figure 4:**
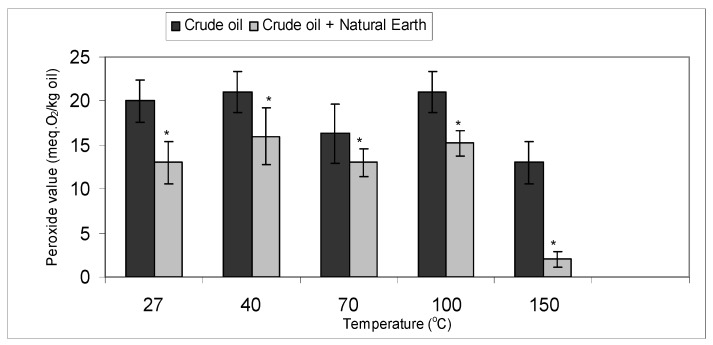
Comparing bleaching temperatures of crude ostrich oil with and without the natural earth clay in removing peroxides.

The controls showed that increasing the bleaching temperature did not necessarily increase the formation of peroxides in the crude ostrich oil, in fact at 150 °C, a reduction in peroxides was observed. Previous studies have shown that at higher temperatures peroxides tend to decompose fast [31,32]. However, it is evident that the addition of natural earth clay does indeed lower the peroxide value most significantly at 150 °C (Figure 4).

### 2.4. Comparing the Vacuum Techniques of Continuous Flow and Nitrogen Trap

**Figure 5:**
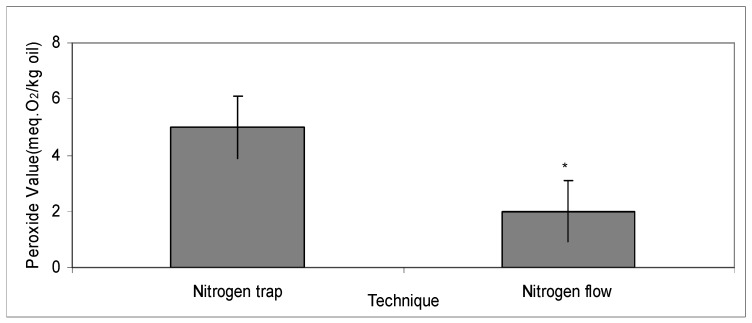
Comparing the vacuum techniques of continuous flow and nitrogen trap in removing peroxides.

It has been shown that if bleaching is carried out under a nitrogen stream there is no formation of peroxide through oxidation of oil and only decomposition and adsorption occurs [27]. In this experiment, two different vacuum techniques, as described in the Experimental, were evaluated for their efficiency to bleach the crude ostrich oil; the continuous nitrogen flow and nitrogen trap (Figure 5).

The continuous flow of nitrogen, which was more effective in lowering peroxide values, was found to be significantly different, with a reduction of peroxide value from 5 (nitrogen trap technique) to 2 (nitrogen flow technique) (p < 0.01) (Figure 5). However, it is advisable to use the nitrogen trap technique for the bleaching of crude ostrich oil as it is a much more economical method and the peroxide value achievable with the nitrogen trap technique was still very low and within the permissible levels.

## 3. Experimental

*Source of Ostrich oil*: Crude Ostrich oil was supplied by Jelita Impian Sdn Bhd. Crude ostrich oil is obtained by slaughtering ostrich birds and removing the adipose and visceral fat from the carcass. The fat is added with water and microwaved at 150 °C at the intervals of 5 minutes for 30 minutes. The oil was bleached using bentonite, natural earth clay and acid activated clays. 

*Source of clays*: All the clays were supplied by Natural Bleach Sdn Bhd (Subang Jaya, Malaysia). Bentonite clay is an impure absorbent aluminium phyllosilicate clay consisting mostly of montmorillonite with two tetrahedral sheets sandwiching a central octahedral sheet. Natural earth clay is classified as a separate group within the phyllosilicates, consisting of parallel sheets of silicate tetrahedra with Si_2_O_5_ mixed along with other weathered minerals. Acid activated clay is usually of a clay of bentonite origin, which has been treated with acid to improve its adsorbing ability [21]. The efficiency of natural earth clay, bentonite and acid-activated clay in removing impurities of ostrich oil was determined, following which the effective duration for the clay to remove the impurities in ostrich oil was also determined. This was done by adding 10% of clay in ostrich oil at room temperature and rotating at constant speed for various durations ranging from 1, 4, 8, 16 and 24 hours. The effective time required to carry out the bleaching process with the lowest peroxide value was determined. The effective temperature required to bleach ostrich oil was examined using bentonite and natural earth clay and by manipulating the temperature from 27 °C up to 150 °C. The most efficient temperature required to reduce peroxide value was determined. Finally, the ostrich oil bleaching process was compared using two different vacuum techniques; nitrogen trap technique (nitrogen gas is trapped using a balloon) and continues nitrogen flow technique (continuous nitrogen gas flow). The efficiency of bleaching ostrich oil as described above was compared by assessing peroxide value using iodometric method according to American oil Chemist Society Method (A.O.C.S. Cd 8-53 Method) as well as PORIM Test Methods [33].

*Bleaching process*: The crude ostrich oil (100 mL) was treated with acid activated, bentonite or natural earth clay (10 g) at room temperature. The oil added with clay was agitated at room temperature for 24 hours. 

*Peroxide value determination*: Once the bleaching process was completed, acetic-acid - chloroform solution (3:230 mL) was mixed with the bleached ostrich oil sample (5 g). The sample was swirled until fully dissolved, after which saturated potassium iodide (0.5 mL) was added using a pipette. The solution was then swirled for 1 minute and distilled water (30 mL) was added, followed by a few drops of starch. The solution was then titrated against 0.01 N sodium thiosulphate solution with constant and vigorous shaking until the yellow iodine color disappeared. The titration point was noted and recorded according to the A.O.C.S. Methods.

The statistical significance was determined using SPSS software and tests conducted were ANOVA and T-Test. 

## 4. Conclusions

An effective and economical method to refine crude ostrich oil was developed in this study. This technique to evaluate the effectiveness of the bleaching process is based on a iodometric method [20]. Contradictory results have been found in some studies with respect to optimizing the bleaching techniques for various oils. Some authors have reported that acid-activated clay is more effective in bleaching oils as compared to other types of clays [10,21]. Our study on the other hand, showed that natural earth clay is far more effective in bleaching crude ostrich oil as compared to acid-activated clay. This could be due to the high pore volume and pore size of natural earth clay which enables sufficient adsorption of trace element impurities as compared to acid activated clay [34]. The minimal duration required to bleach ostrich oil was observed to be 1 hour. Our results support the findings of other workers (palm oil, 30 min [21], cotton oil, 120 min [35] and soy oil 30 min [28]). A significant difference of our findings is the effective temperature required to bleach ostrich oil. Previous studies have shown the effective temperature to bleach oils to be around 90 °C to 100 °C [21,28]. In this study, the effective temperature was established to be 150 °C. Although the effective temperature required to bleach ostrich oil is high, we observed that the percentage of peroxide reduction (90%) at this temperature was significant, with a peroxide reduction to a value of 2 after bleaching at 150 °C, while crude oil had peroxide value of 20, whereas bleaching at 90 °C and 100 °C only saw a peroxide reduction to 15 and 13 respectively. This was proven in our next study where we compared the efficiency of natural earth clay in bleaching ostrich oil and crude ostrich oil (without clay) at various temperatures and the result showed natural earth clay was very efficient in bleaching ostrich oil at 150 °C as compared to other temperatures. In the final part of this research, two vacuum techniques were compared, namely the nitrogen trap technique and the continuous flow technique. Although our results showed that nitrogen flow technique (peroxide value = 2) was more efficient than nitrogen trap technique (peroxide value = 5), both resulted in minimal peroxide values, therefore in this study, the nitrogen trap technique is recommended as it is more cost effective and was observed to be an odourless process, as compared to the nitrogen flow technique. This study therefore confirms that an effective and economical method to bleach crude ostrich oil requires the use of 10% natural earth clay at 150 °C for 1 hour using the nitrogen trap vacuum technique. This method bleaches ostrich oil successfully, removes peroxides and other toxins but maintains the essential component of the oil which is the omega 3, omega 6 and omega 9 fatty acids (unpublished results). These findings are essential in the processing of the ostrich oil to ensure its quality if it is intended to be used for therapeutic purposes.

## Figures and Tables

* designates a significant difference from control (without clay) (P < 0.01).

* designates a significant difference between clays using bentonite clay as control (P < 0.01).

* designates a significant difference between clays using bentonite clay as control (P < 0.01).

* designates a significant difference from control (crude oil only) (P < 0.01).

* designates a significant difference from control (nitrogen trap technique) (P < 0.01).
